# Cloning and Characterization of a New Chitosanase From a Deep-Sea Bacterium *Serratia* sp. QD07

**DOI:** 10.3389/fmicb.2021.619731

**Published:** 2021-02-24

**Authors:** Qiuling Zheng, Xiangjun Meng, Mingyang Cheng, Yanfeng Li, Yuanpeng Liu, Xuehong Chen

**Affiliations:** ^1^Department of Pharmacology, School of Basic Medicine, Qingdao University, Qingdao, China; ^2^Qingdao Mental Health Center, Qingdao, China

**Keywords:** chitosanase, chitooligosaccharides, deep-sea bacterium, *Serratia* sp. QD07, fermenter

## Abstract

Chitosanase is a significant chitosan-degrading enzyme involved in industrial applications, which forms chitooligosaccharides (COS) as reaction products that are known to have various biological activities. In this study, the gene *csnS* was cloned from a deep-sea bacterium *Serratia* sp. QD07, as well as over-expressed in *Escherichia coli*, which is a new chitosanase encoding gene. The recombinant strain was cultured in a 5 L fermenter, which yielded 324 U/mL chitosanases. After purification, CsnS is a cold-adapted enzyme with the highest activity at 60°C, showing 37.5% of the maximal activity at 0°C and 42.6% of the maximal activity at 10°C. It exhibited optimum activity at pH 5.8 and was stable at a pH range of 3.4–8.8. Additionally, CsnS exhibited an endo-type cleavage pattern and hydrolyzed chitosan polymers to yield disaccharides and trisaccharides as the primary reaction products. These results make CsnS a potential candidate for the industrial manufacture of COS.

## Introduction

Chitosan, the primary deacetylation product of chitin, is a linear cationic polysaccharide comprising of β-(1,4)-linked D-glucosamine (GlcN or D unit) and *N*-acetyl-D-glucosamine (GlcNAc or A unit) (Muanprasat and Chatsudthipong, 2017). The chitosan polymer is a natural alkaline polysaccharide, insoluble in most neutral liquids such as water (Naveed et al., 2019). Chitooligosaccharide (COS), the degradation product of chitosan, has aroused an increasing interest due to its excellent biological properties and potential applications. Of particular importance are its admirable biological activities including anti-microbial (Fernandes et al., 2008), anti-fungal (Mei et al., 2015), anti-oxidant (Ma et al., 2020), anti-inflammatory (Kunanusornchai et al., 2016), anti-obesity (Huang et al., 2015), anti-tumor (Suzuki et al., 1986; Zhao et al., 2019), anti-hypertensive (Huang et al., 2005), anti-HIV-1 (Artan et al., 2010), anti-Alzheimer’s (Eom et al., 2013), and immune-enhancing effects (Zhang et al., 2014). It also shows promise as a drug/DNA delivery agent (Kumari et al., 2018; Li et al., 2019a; Wang et al., 2020). Therefore, COS is a promising candidate with potential application in several fields, including the medical and pharmaceutical industries.

Chitosanases (EC 3.2.1.132) are divided into five families: GH-5, GH-8, GH-46, GH-75, and GH-80 in the CAZy database (Thadathil and Velappan, 2014). Families GH-5 and GH-8 could hydrolyze chitosan and some other glycosides, while the families GH-46, GH-75 and GH-80 comprise chitosanases merely (Johnsen et al., 2010). Chitosanases catalyzed the breaking of the β-(1,4) glycosidic bonds in chitosan to yield low molecular weight chitosan (LMWC) or COS with different Degree of Polymerization (DP) (Brzezinski, 2011). Due to the substrate specificity of different chitosanases, the degradation of COS by different DP resulted in different biological activities. For example, the DP 1-3 inhibits matrix metalloproteinase-9 in human fibrosarcoma cells (Van Ta et al., 2006); the DP 2-6 (Qiao et al., 2011) exhibit anti-inflammatory activity; the DP6 shows anti-tumor activity (Xiong et al., 2009), whereas the DP 8-12 have higher anti-oxidant activity (Li et al., 2012).

Currently, COS can be produced by chemical, physical, electrochemical and enzymatic methods (Liang et al., 2018) Owing to the lack of contaminants and the easily controllable nature of the process, using chitosanases are relatively satisfactory tools to produce COS in enzymatic methods. Considering the lack of a suitable commercial chitosanase for the production of COS, we previously established an efficient affinity purification method to rapidly detect chitosanase in bacterial cultural supernatant (Li et al., 2019b), and several chitosanase-producing marine bacteria strains including a deep-sea bacterium *Serratia* sp. QD07 were successfully isolated. Chitosanase is mainly derived from bacteria (Luo et al., 2020), but to a lesser extent from fungi (Wang et al., 2008), plant tissues (Osswald et al., 1994) and viruses (Blanc et al., 2014). Although many chitosanases have been reported, there are only a few chitosanases with cold adaptability (Johnsen et al., 2010; Qin et al., 2018; Yang et al., 2019). The yield of chitosanase of microorganisms reported so far is different.

In this study, the gene *csnS* was cloned from *Serratia* sp. QD07, as well as expressed in *Escherichia coli*, which is a chitosanase encoding gene. Additionally, an efficient fermentation and purification method was established and the biochemical features of CsnS were characterized.

## Results and Discussion

### Sequence Analysis of CsnS

The deep-sea bacterium *Serratia* sp. QD07 was isolated from a sample of sea mud in the South China Sea (depth: 1179 m). This strain proliferated rapidly in the selection medium containing chitosan [0.5% (w/v)] and showed high chitosanases activity. Results of the sequence analysis showed that its genome contained a putative chitosanase-encoding gene, *csnS*. In this study, the *csnS* gene was cloned from *Serratia* sp. QD07, which consisted of an intact open reading frame (ORF) of 756 base pairs, and an encoded protein, CsnS containing 251 amino acid residues. Signal peptide analysis revealed that the *N*-terminal of CsnS lacked signal peptide. The theoretical pI of CsnS was 5.56, as well as the molecular weight (MW) of CsnS was 27.1 kDa.

**FIGURE 1:**
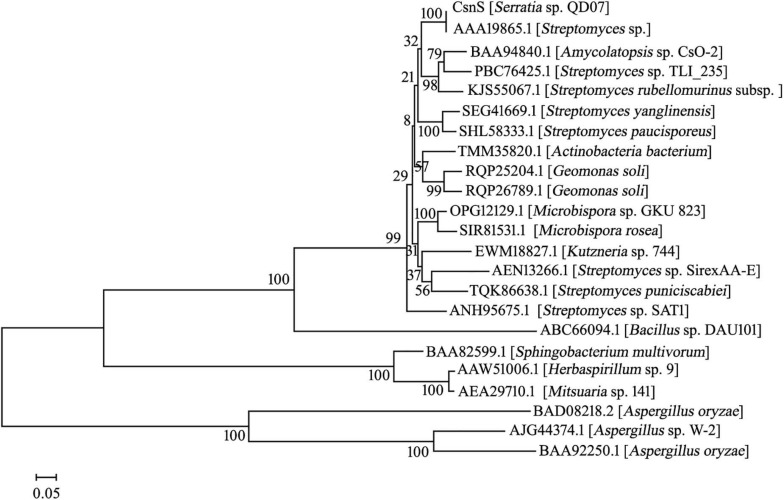
The phylogenetic relationships between CsnS and other chitosanases from GH family 46, 75, and 80 is shown in the neighbor-joining tree. The bootstrap test of the tree repeated 1,000 times.

**FIGURE 2:**
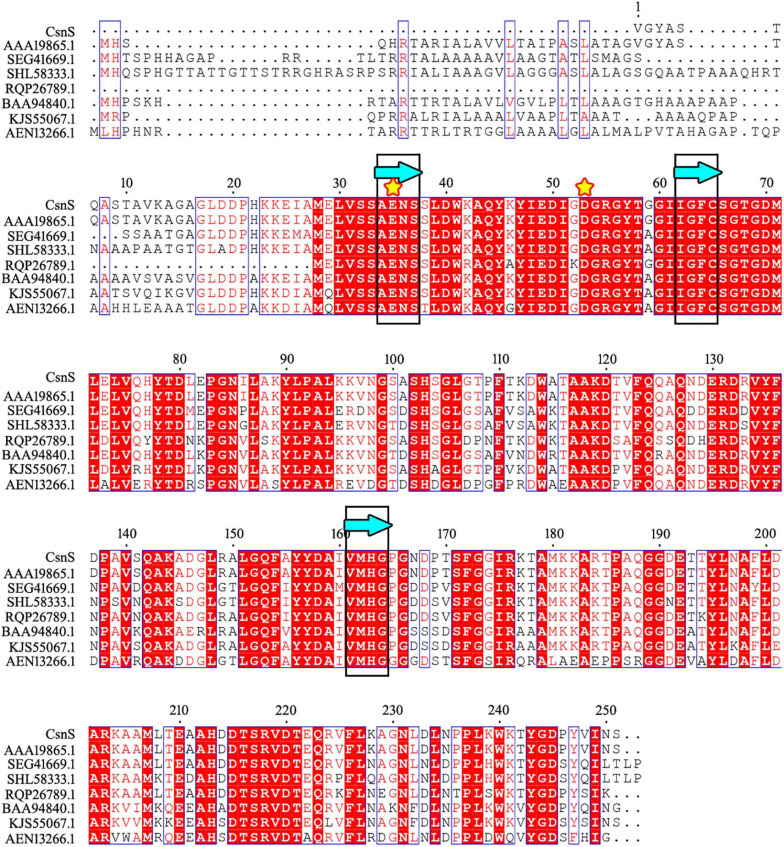
Comparison of the sequence of CsnS with reported chitosanase activity from the GH-46 family: chitosanase from *Streptomyces* sp. N174 (GenBank number: AAA19865.1); chitosanase from *Streptomyces yanglinensis* (GenBank number: SEG41669.1); chitosanase from *Streptomyces paucisporeus* (GenBank number: SHL58333.1); chitosanase from *Geomonas soli* (GenBank number: RQP26789.1); chitosanase from *Amycolatopsis* sp. CsO-2 (GenBank number: BAA94840.1); chitosanase from *Streptomyces rubellomurinus subsp. indigoferus* (GenBank number: KJS55067.1); chitosanase from *Streptomyces* sp. SirexAA-E (GenBank number: AEN13266.1). The same sugar binding and catalytic sites are marked with blue bands (framed in black) and yellow stars, respectively.

The phylogenetic tree was established by sequences analysis of CsnS and some other reported chitosanases from families GH-46, GH-75, and GH-80 (Figure 1). CsnS was inferred to belong to the glycoside hydrolyase-46 (GH-46) family. It was found that CsnS showed higher homology with a GH-46 chitosanase (GenBank: AAA19865.1), obtained from a species of the genus *Streptomyces*. In addition, the conservative domain database (CDD) search was conducted on NCBI, and CsnS was a recognized chitosanase with a conservative domain, belonging to the family GH-46. To further analyze its structure, multiple sequence alignment (MSA) between CsnS and seven other GH-46 chitosanases was established. Results from the MSA (Figure 2) showed that the enzyme contains some conserved regions with this chitosanase of family GH-46. The conserved sites Glu35 and Asp53 play vital roles in the hydrolysis of glycosides (Saito et al., 1999; Lyu et al., 2015). The regions ‘AENS’ (34–37), ‘IGFC’ (62–65), ‘VMHG’ (161–164), and sites Tyr47, Ile51, Asp53, Arg55, Thr58, Asp69, and Tyr125 are related to sugar binding (Lyu et al., 2014).

### Fermentation and Purification of CsnS

**FIGURE 3:**
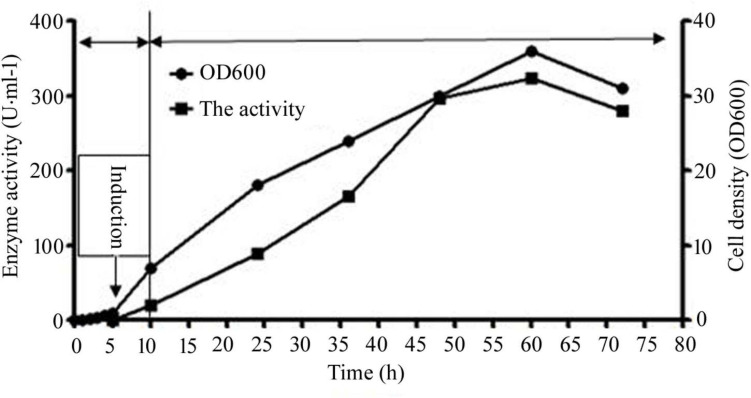
The extracellular activity and cell growth of CsnS production by cultivation. And the entire culture procedure was distributed to three phases: the growth cultivation phase, the induction cultivation phase, and the fed-batch cultivation phase. Since OD 600 reached 1, the temperature was dropped to 20°C, as well as IPTG (0.1 mM) was added. Subsequently, the original glycerol was absolutely consumed, the feeding phase of fed-batch cultivation was initiated.

The first phase of the process commenced with an original glycerol concentration of 10 g/L. At the beginning of the second stage, when OD 600 value was 1.0, IPTG (0.1 mM) was added, and the fermentation temperature was reduced to 20°C. The culture volume used for expression and purification is 50 mL. During the entire process, the feeding was controlled by maintaining the concentration of dissolved oxygen at 30% of air saturation. Once the enzyme activity decreased, the fermentation process was stopped (Figure 3). At the end of the process at 60 h, the extracellular CsnS activity was calculated to be 324 U/mL.

**FIGURE 4:**
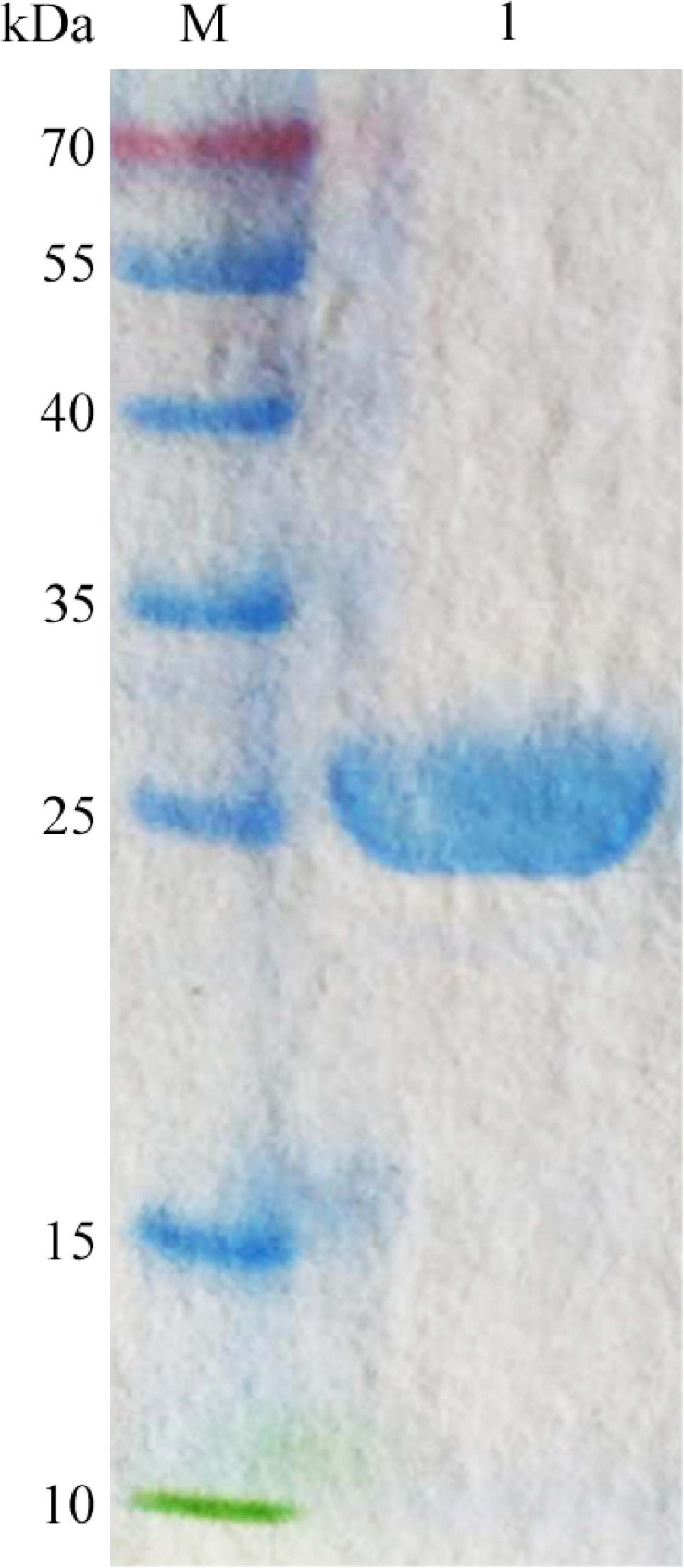
SDS-PAGE analysis of the purified CsnS. M, molecular weight markers; 1, purified CsnS.

The recombinant CsnS was purified 1.7-fold using Ni-NTA-Sepharose column chromatography. Its MW was determined by sodium dodecyl sulfate-polyacrylamide gel electrophoresis (SDS-PAGE) to be about 27 kDa (Figure 4), corresponding to the predicted MW. The specific activity of purified CsnS was determined to be 412.6 U/mg. The activity recovery was 91.9%.

### The Different Conditions on the Production of CsnS

#### Effects of Different Cell Density OD 600 on the Induction Cultivation Phase on CsnS

Cell density OD 600 was a critical parameter, which led to a significant effect on the extracellular activity at induction cultivation phase. As shown in Supplementary Figure 1, the induced cells reached 8 and the extracellular activity was 26 U/mL at OD 600. Whereas, when the induced cells reached 1, the extracellular activity was the highest, 81 U/mL, which was 3.1-fold higher than that of cell density (OD 600) at 8. Moreover, it was 2.1-fold higher than that at 4 of OD 600, which exhibited an extracellular activity of 38 U/mL. The extracellular activity was closer to cell density OD 600 at 1 and 2, after induction, and the extracellular activity of cell density OD 600 at 2 was 71 U/mL which was lower than OD 600 at 1. Consequently, at OD 600 of the induced cells reached 1 of OD 600, *E. coli* was more beneficial to the secretion expression.

#### Effects of Temperature on the Secretion of CsnS

Temperature is a key parameter in the production of recombinant proteins in *Escherichia coli*. Generally, the recombinant *E. coli* would grow slowly at low temperature, and low production rate always conduced low protein yields. However, the high temperature would denature recombinant proteins and reduce enzyme activities. As shown in Supplementary Figure 2, under the induction temperature of 35°C, the number of OD 600-induced cells attained 8.9, while the extracellular activity was only 38 U/mL. By contrast, under the induction of 20°C, the coordination between protein synthesis and translocation was better, and extracellular activity was the highest, 82 U/mL, which was 2.1-fold higher than that under the induction of 35°C. The extracellular activity was 58 U/mL, 1.4-fold higher than that of 30°C. This high yield might be due to the low temperature which reduced the denaturation rate of the target proteins and allowed the freshly synthesized peptides to fold correctly. Although 15°C might be more suitable for CsnS production, the significantly lowered cell density limited its total protein yield. Consequently, *E. coli* was more favorable for secretion expression at a lower temperature of 20°C.

#### Effects of Concentration of IPTG on the Secretion of CsnS

The IPTG was added to induce the protein of CsnS expression. Actually, the IPTG was potentially toxic chemical that restrained cell growth with increasing concentration. Experiments were performed to investigate and identify the optimal induction concentration of IPTG. As shown in Supplementary Figure 3, at an induction concentration of 0.8 mM, OD 600 of the induced cells reached only 6.1, but the extracellular activity was also low and only 36 U/mL. When the induced concentration was 0.1 mM, the extracellular activity was the highest, 86 U/mL, 2.3-fold higher than that of 0.8 mM. Moreover, this activity was 1.4-fold higher than that of 0.4 mM, which exhibited the extracellular activity of 60 U/mL. The extracellular activity was closer at induction concentrations of IPTG at 0.1 mM and 0.2 mM, and the extracellular activity of induction concentrations at 0.2 mM was 77 U/mL. This low extracellular activity might be due to the low concentration of IPTG at 0.05 mM and high concentration of IPTG at 0.8 mM, which causes an inhibitory effect on the growth of *E. coli.* Consequently, at induction concentration of 0.1 mM, *E. coli* was more beneficial to the secretion expression.

#### Biochemical Properties of CsnS

**FIGURE 5:**
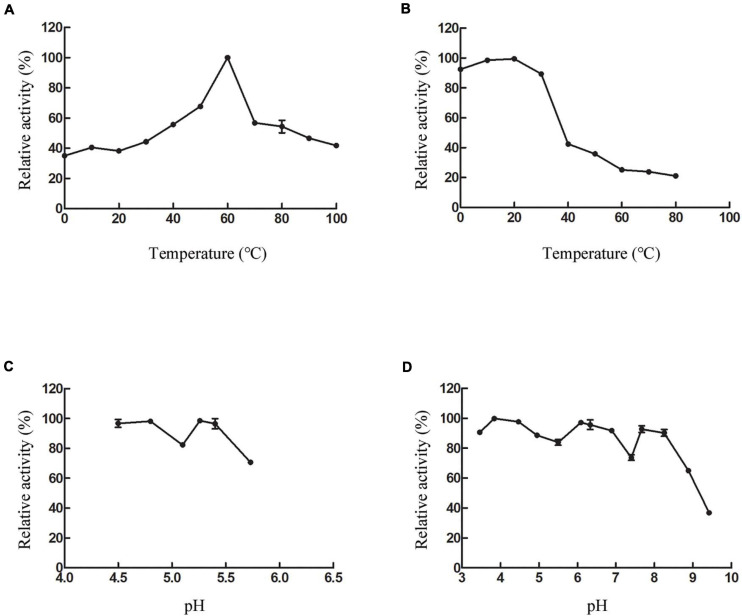
Effect of temperature and pH on CsnS activity. **(A)** Effect of temperature on the activity of CsnS. **(B)** Thermal stability of CsnS. Enzyme was incubated for 2 h at 0–80°C. **(C)** Effect of pH on the activity of CsnS (Sodium acetate buffer pH 4.5–5.73). **(D)** pH stability of CsnS. The pH stability was analyzed by measuring the residual activity after pretreating the enzyme in different buffers at 4°C for 48 h (citric acid/Na2HPO4 buffer, pH 2.9–6.09; Na2HPO4/NaH2PO4 buffer, pH 6.33–7.40; Tris-HCl buffer, pH 7.67–8.25; glycine-NaOH buffer, pH 8.88–9.42). Data represent mean ± SD from three independent experiments.

The biochemical properties of recombinant CsnS were determined by the purified enzyme. As shown in Figure 5A, at 60°C, CsnS showed the highest activity. At the same time, CsnS exhibits 37.5% and 42.6% of its maximal activity at 0°C and 10°C, respectively, which is a cold-adapted enzyme. It can retain approximately 40% of enzyme activity at temperatures ranging from 0 to 100°C, which indicates the ruggedness of CsnS in extreme temperature conditions. Compared with temperature-sensitive enzymes, this kind of temperature-resistant enzyme has certain advantages in industrial production. On the one hand, the preparation cost of this kind of enzyme preparation is reduced, and the enzyme preparation has high stability, which can maintain the activity for a long time. On the other hand, the requirements for the reactor cooling system are reduced, which reduces energy consumption and pollution. In addition, CsnS has good enzyme activity at 60°C, and there are few hybrid bacteria living at 60°C, thus reducing the contamination of bacterial metabolites to products and improving the purity of products. After incubation at temperatures between 0 and 30°C for 2 h, the enzyme retained more than 80% of the initial activity (Figure 5B). These results show that the enzyme can be stored at room temperature and is capable of catalyzing hydrolysis at this temperature. This is a desirable property in industrial applications.

Assays to determine the optimal pH for CsnS activity were carried out with 0.3% (w/v) chitosan at pH 4.5–5.7. CsnS retained more than 60% of its activity in the experimental range and displayed optimal activity at pH 5.2 (Figure 5C). Additionally, the enzyme can retain more than 80% activity at pH 4.5–5.4. In order to study pH-stability of the enzyme, various buffers ranging from pH 3.4–9.4 were used in the assays (Figure 5D). The enzyme was found to be stable and retained more than 70% of its initial activity at a pH range of 3.4–8.2. At a higher pH value of 9.4, it retained about 35% of its original activity. Therefore, CsnS demonstrates high catalytic properties in either an acidic or alkaline environment and is stable over a broad range of pH. Because the fermentation process of pH sensing electrode sensor has been delayed, the pH of the fermentation medium is not fixed during the enzyme production process. Fortunately, pH has little effect on bacterial growth. Thus, pH fluctuations do not affect the enzyme activity of chitosanase during the fermentation process.

The effect of metal ions and reagents on the activities of CsnS was shown in Table 1. We found that except Cu^2+^ and Ni^2+^, most metal ions can promote CsnS activity and maintain it above 70%. CsnS shows a strong ability to resist the interference of metal ions. Since most industrial equipments are constructed with metal, the non-reactivity of CsnS to metal ions could facilitate its use in the production process, thereby expanding its application efficiency.

**TABLE 1:** Effects of metal ions and reagents on the activity of CsnS.

**Reagent added**	**Concentration (mM)**	**Relative activity (%)**
None	–	100.0 ± 0.0
MgSO4	20	138.4 ± 3.3
FeCI_3_	20	127.0 ± 3.1
BaCI_2_	20	138.0 ± 4.4
ZnCI_2_	20	114.3 ± 2.8
EDTA	20	131.4 ± 1.6
FeSO_4_	20	140.3 ± 2.6
KCI	20	135.4 ± 0.1
CuSO_4_	20	21.5 ± 0.4
Li_2_SO_4_	20	139.7 ± 3.1
CoCI_2_	20	96.3 ± 3.8
SDS	20	119.3 ± 0.7
(NH_4_)_2_SO_4_	20	141.4 ± 4.1
Al_2_(SO_4_)_3_	20	140.7 ± 0.8
CaCI_2_	20	139.2 ± 3.6
Nicl2	20	70.5 ± 0.8

*Activity without addition of chemicals was defined as 100%. Data are shown as means ± SD (*n* = 3).*

#### Action Mode and Reaction Product Analysis

**FIGURE 6:**
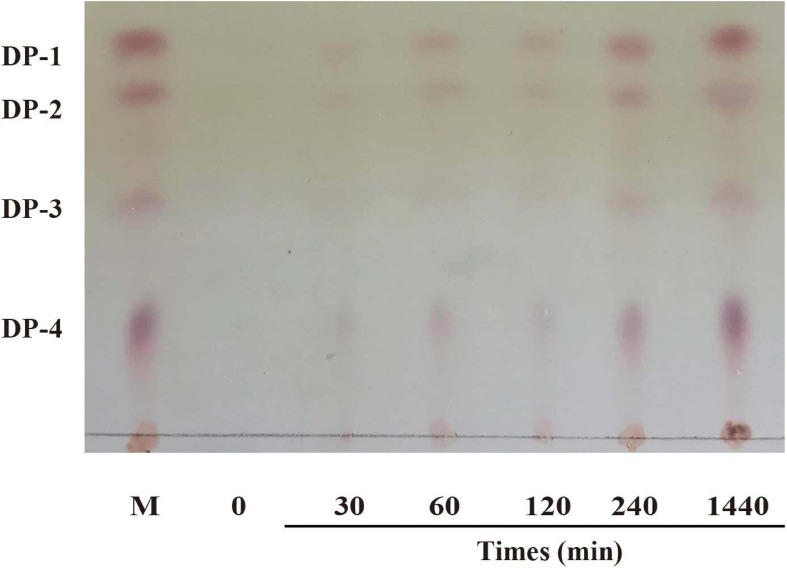
TLC analysis of products after enzymatic hydrolysis.

Thin-layer chromatography (TLC) was used to analyze the reaction products and the action modes of CsnS. As shown in Figure 6, no obvious chitooligosaccharides products appeared during the first 30 min of the reaction. When the substrate was hydrolyzed for 60 min, reaction products were observed, which were mainly a mixture of disaccharides (DP2), trisaccharides (DP3) and tetrasaccharides (DP4). With the extension of reaction time, the relative ratio of DP2 and DP3 oligomers enhanced, whereas those of the higher DP products reduced. Compared with CsnM (Zhou et al., 2019) we studied previously, the product increased DP-1 and DP-4. According to literature reports, DP2-4 chitosan oligosaccharide has a promising potential for a wide range of applications in the medical field, by virtue of its anti-inflammatory and anti-bacterial activities. These reaction products were similar to most known chitosanases, such as the CsnB from *Bacillus* sp. BY01 (Yang et al., 2019), CsnQ from *Bacillus* sp. Q1098 (Ma et al., 2020), Csn-BAC from *Bacillus* sp. MD-5 (Yang et al., 2020), and Csn-SP from *Bacillus* sp. DAU101 (Lee et al., 2006 and Table 2), all of which produced (GlcN)_2_ and (GlcN)_3_ as final products. Till date, the main products of chitosanases reported in the literature are mixtures of DP3-DP8, such as chitosanase from *Bacillus mycoides* TKU038 (Liang et al., 2016) and *Bacillus* sp. strain KCTC 0377BP (Choi et al., 2004), which are difficult to separate. We foresee CsnS as a potentially valuable agent in the manufacture of easily separable products in industrial production.

**TABLE 2:** Comparison of the CsnS properties with other reported chitosanases.

**Name**	**GenBank no.**	**Orgnism source**	**Optimal pH**	**Stable pH range**	**Optimal temperature (°C)**	**Products (DP)**	**References**
CsnS	MN963773	*Serratia* sp. QD101	5.8	3.4-8.8	60	2-3	This study
CsnQ	MN963773	*Bacillus* sp. Q1098	5.3	6.8-9.1	60	2	Mei et al., 2015
CsnB	MN531545	*Bacillus* sp. BY01	5.0	4.6-5.8	35	2-3	Liang et al., 2018
Csn-BAC	CP021911	*Bacillus* sp. MD-5	7.0	6.0-8.0	40	2-3	Li et al., 2019a
CS038	–	*Bacillus mycoides* TKU038	6.0	4.0-10.0	50	3-9	Lyu et al., 2015
CSN-SP	DQ316095	*Bacillus* sp. DAU101	7.5	−	50	2-3	Saito et al., 1999
chitosanase	AF334682	*Bacillus* sp. strain KCTC 0377BP	5.0	4.0-8.0	60	3-8	Luo et al., 2020
chitosanase	GQ487532	*Janthinobacterium* sp. 4239	5.0	−	45	1-2	Wang et al., 2020

## Materials and Methods

### Materials and Bacterial Cultures

We purchased *E. coli* strains BL21 (DE3) as well as expression vector, pET22b (+) from Takara (Dalian, China). From Aladdin Biochemical Technology Co., Ltd., (Shanghai, China), we purchased chitosan (degree of deacetylation ≥ 95%, viscosity: 100–200 mpa⋅s). And from Merck (Darmstadt, Germany), we purchased the thin-layer chromatography (TLC) silica gel plates.

### Isolation of the Bacterial Strains *Serratia* sp. QD07

Deep-sea mud samples were collected from South China Sea (depth 1,179 m, E 118.16° N 22.01°) in August 2017. The samples were immersed, diluted, and spread on 2216E medium. Isolated colonies were cultured on chitosan selective medium containing 0.5% (w/v) chitosan, 0.1% (w/v) KH2PO4, 0.2% (w/v) K2HPO4, 0.07% (w/v) MgSO4, 0.1% (w/v) NaCl, 0.01% (w/v) CaCl2, and 1% (w/v) agar. Strain QD07 was isolated and stored in our laboratory. Furthermore, this strain was identified as *Serratia* sp. QD07 using 16S rRNA gene sequence analysis (Genbank number: MT576555).

### Sequence Analysis

The draft genome of *Serratia* sp. QD07 was determined by using our 2nd generation sequencer. A fictitious chitosanase encoding the gene *csnS* was identified and its protein sequence was registered in the GenBank database (Genbank number: MT241387). To further analyze the *csnS* gene sequence, we used the ORF search program^1^ to determine the open reading frame (ORF). The signal peptide of CsnS via the SignalP 5.0 server^2^ was analyzed. To improve phylogenetic analysis, using the Conserved Domain Database (CDD) to obtain domain and family information. For multi-sequence alignment, we used ClustalX2.1 and ESPript^3^. The phylogenetic tree was constructed by the MEGA 7.0 bootstrap adjacency binding method. In addition, it was using ExPASy^4^ to determine the theoretical isoelectric point (pI) and MW of CsnS.

### Expression of Recombinant Chitosanase

A polymerase chain reaction (PCR) was used to amplify the CsnS fragment without a terminator. The PCR primers used were Ep-CsnS-F and Ep-CsnS-R, which contained the recognition sites *Nco*I and *Xho*I, respectively. The amplified sequence was inserted into plasmid pET22b between the same recognition sites. *Escherichiacoli* BL21 (DE3) containing the recombinant plasmid pET22b-CsnS plasmid was inoculated into Luria-Bertani (LB) broth for growth at 37°C for 6 h.

### High-Density Fermentation

60% glycerol stocks of the *E. coli* BL21-pET22b-CsnS cells were streaked on LB solid medium culture panel supplemented with 50 μg/mL ampicillin and incubated for 26 h at 37°C. *E. coli* colonies containing recombinant plasmid were inoculated in 5 mL LB medium containing 50 mg/mL ampicillin as well as incubated in a rotating shaking table (180 rpm) at 37°C overnight. The overnight cultivation (0.5 mL) was diluted to 50 mL Terrific Broth Medium (TB) including 50 mg/mL ampicillin and cultivated on a rotating shaking table (180 rpm) at 37°C before the optical density reading at 600 nm (OD 600) was 8.0. Accordingly, it was cultured in a bioreactor and fermented in a 5 L fermentation tank (BL Bio-5GJ, Shanghai, China). The 5% (v/v) seed culture was inoculated into TB medium. The complete culture process was divided into three stages. At the beginning of the first stage, the initial concentration of glycerol was 10 g/L at 37°C. When the OD 600 reading was 1.0, we moved to the second phase in which, isopropyl β-d-thiogalactoside (IPTG, 0.1 mM) was added to induce the protein formation. The cultivation temperature was decreased to 20°C and the sample was incubated. When glycerol in the medium was completely consumed, as indicated by a sudden increase in dissolved oxygen (DO) and it was reaching 60% of air saturation, we commenced the third phase. In this phase, we continued fermentation as a fed-batch with controlled feeding to maintain a constant concentration of DO at 30% of air saturation. In the entire procedure, the pH was maintained at 7.0 throughout the addition of 20% (v/v) ammonia solution. The speed of cascade impeller was kept between 300 and 500 rpm, and the air source used compressed air with an airflow of 6 vvm.

### Purification of Recombinant CsnS

The crude enzyme in the supernatant was harvested and centrifuged at 13,000 *g* at 4°C for 15 min. Phosphate buffers A (pH 8.0, 500 mM NaCl) and B (pH 8.0, 500 mM NaCl, 500 mM iminazole) were used in the purification process. Before the crude enzyme was loaded onto an Ni-NTA column in the AKTA150 automatic purification system, five column volumes of buffer A were used to wash the column in order to enable it to load proteins effectively. The sample loading speed was maintained at 1 mL/min. The target enzyme was eluted with buffer B by gradient elution. The enzyme purity and molecular mass were determined using SDS-PAGE.

### The Fermentation Process of CsnS

In order to further optimize the fermentation, the recombinant strains containing CsnS were selected. To enhance the extracellular production of recombinant CsnS, we developed the process optimization strategies, as well as the effects of induction culture steps at various cell densities OD 600, induction temperatures, and various concentrations of IPTG were investigated.

#### Effects of Different Cell Density OD 600

To optimize the cell density OD 600 of induction for CsnS production, *Ecoli* strain harboring CsnS were induced at 0.5,1, 2, 4, and 8 in TB medium, respectively. The cell was incubated in 50 mL TB including 50 mg/mL ampicillin and cultured on a rotary shaker (180 rpm) at 37°C. When the optical density reached at various 600 nm (OD 600), and then reduced the temperature to 20°C. Accordingly, IPTG (0.1 mM) was added to induce the expression of target protein, and the cells were incubated for a further 24 h at 20°C sequentially.

#### Effects of Induction Temperature

To optimize the induction temperature of CsnS, *E.coli* strain containing CsnS were induced in TB medium at 15, 20, 25, 30, and 35°C, respectively. The cell was incubated in 50 mL TB including 50 mg/mL ampicillin, as well as cultured on a rotary shaker (180 rpm) at 37°C until the optical density at 600 nm (OD 600) attained 1. Accordingly, we put the shake flasks in a rotary shaker at various induction temperatures. Accordingly, IPTG (0.1 mM) was added to induce the expression of target protein, as well as the cultivation was continued for another 24 h at 20°C.

#### Effects of Different Concentrations of IPTG

The cells were incubated in 50 mL TB including 50 mg/mL ampicillin and placed in a rotary shaker (180 rpm) at 37°C and 600 nm (OD 600), until the optical density reached 1, and then dropped the temperature to 20°C. Various concentrations of IPTG (0.05, 0.1, 0.2, 0.4, and 0.8 mM) were added to induce the expression of the target protein, and the incubation at 20°C for an additional 24 h sequentially.

### Assay of CsnS Activity

It was using the dinitrosalicylic acid method (DNS) to analyze CsnS activity. After diluting the enzyme in sodium acetate buffer (50 mM, pH 5.8), the enzymatic activity was measured using 100 μL enzyme and 900 μL 0.3% (w/v) chitosan at 60°C for 10 min. Next, 0.75 mL of 3,5-dinitrosalicylic acid was added into the mixture to reduce the reaction. Then, the mixture was boiled for 10 min and centrifuged to remove the debris, as well as the OD was determined at 520 nm. The mixture with inactivated enzyme constituted the control. One unit (U) of chitosanase was defined as the amount of chitosanase that delivered 1 μmol of reducing sugars per min under optimal conditions.

### Effect of Temperature, pH and Metal Ions on CsnS

The enzyme was diluted 200 times with phosphate buffer (pH 7.0) for biochemical characteristic assays. The substrate chitosan 0.3% (w/v) at pH 5.8 was used in the assays to evaluate the influence of temperature and metal ions on the enzyme. The optimal reaction temperature of CsnS was determined, and its activity was measured at various reaction temperatures ranging from 0 to 100°C. Thermal stability was investigated by measuring the residual activity after pre-incubating the purified enzyme at 0–80°C for 2 h. In order to evaluate the effect of metal ions on CsnS, several solutions of metal ions at a final concentration of 20 mM solution were incubated with the purified enzyme at 4°C for 48 h and the enzyme activity was measured. To determine the optimal pH, the substrate chitosan (0.3% w/v) at varying pH (4.50–5.73) was prepared in sodium acetate buffer. Finally, to determine the effect of pH, the purified enzyme was incubated in various buffers with pH levels ranging from 3.4 to 9.4.

### Analysis of CsnS Degradation Products

We used TLC to detect degradation products of CsnS, Purified CsnS (50 μL) was added to 450 μL of chitosan solution (3 mg/mL) and reacted at 60°C. The samples were subjected to several reaction times (10, 30, 60, 120, 240, and 1,440 min). The reaction was terminated by boiling the sample for 10 min. The inactivated samples (2 μL) were spotted on to a TLC plate and developed with *n*-propanol/30% ammonia (2:1) solution. The plates were sprayed with 0.5% ninhydrin in ethanol, and heated at 100°C for 20 min to visualize sugars.

## Conclusion

In this study, CsnS, a new member of GH-46 chitosanase from deep-sea bacterium *Serratia* sp. QD07 was cloned as well as over-expressed in *E. coli*. The extracellular production of recombinant CsnS in a 5 L fermenter indicated a high enzyme activity of 324 U/mL. CsnS is a cold-adapted enzyme, which showed the highest activity at 60°C, and exhibited 37.5 and 42.6% of its maximal activity at 0°C and 10°C, respectively. Hydrolytic product analysis indicated that CsnS is an endo-type chitosanase, converting chitosan to (GlcN)_2_ and (GlcN)_3_. Therefore, we can conclude that CsnS has suitable properties and may have potential applications in the production of bioactive COS in the food and pharmaceutical industries.

## Data Availability Statement

The original contributions presented in the study are included in the article/Supplementary Material, further inquiries can be directed to the corresponding author/s.

## Author Contributions

QZ: formal analysis, writing-original draft preparation, conceptualization, methodology. XM: editing, analysis, data curation, and software. MC: analysis. YL: software. YL: editing. XC: funding acquisition, supervision. All authors contributed to the article and approved the submitted version.

## Conflict of Interest

The authors declare that the research was conducted in the absence of any commercial or financial relationships that could be construed as a potential conflict of interest.

## Footnotes

1 https://www.ncbi.nlm.nih.gov/orffinder/

2 http://www.cbs.dtu.dk/services/SignalP/

3 http://espript.ibcp.fr/ESPript/cgi-bin/ESPript.cgi

4 https://web.expasy.org/cgi-bin/protparam/protparam

## Supplementary Material

Supplementary Figure 1 Effects of different cell density OD600 in the induction cultivation phase on the extracellular activity in the shake flasks with TB medium at 0.5,1, 2, 4, and 8.

Supplementary Figure 2 Effects of temperature on the extracellular activity and cell growth *Escherichia coli* strain which was grown in the shake flasks with TB medium at 15,20, 25, 30, and 35°C.

Supplementary Figure 3 Effects of different concentrations of IPTG on the extracellular activity and cell growth in *Escherichia coli* strain which was grown in the shake flasks with TB medium at 0.05, 0.1, 0.2, 0.4, and 0.8 mM.
