# Supplementary material for: Cloning and Characterization of a New Chitosanase From a Deep-Sea Bacterium Serratia sp. QD07
Source: Front Microbiol. 2021 Feb 24;12:619731. doi: 10.3389/fmicb.2021.619731 (PMC7943732; doi:10.3389/fmicb.2021.619731)
Supplement: Supplementary Figure 1 — Effects of different cell density OD600 in the induction cultivation phase on the extracellular activity in the shake flasks with TB medium at 0.5,1, 2, 4, and 8. [file Data_Sheet_1.docx]

Supplementary Material

**
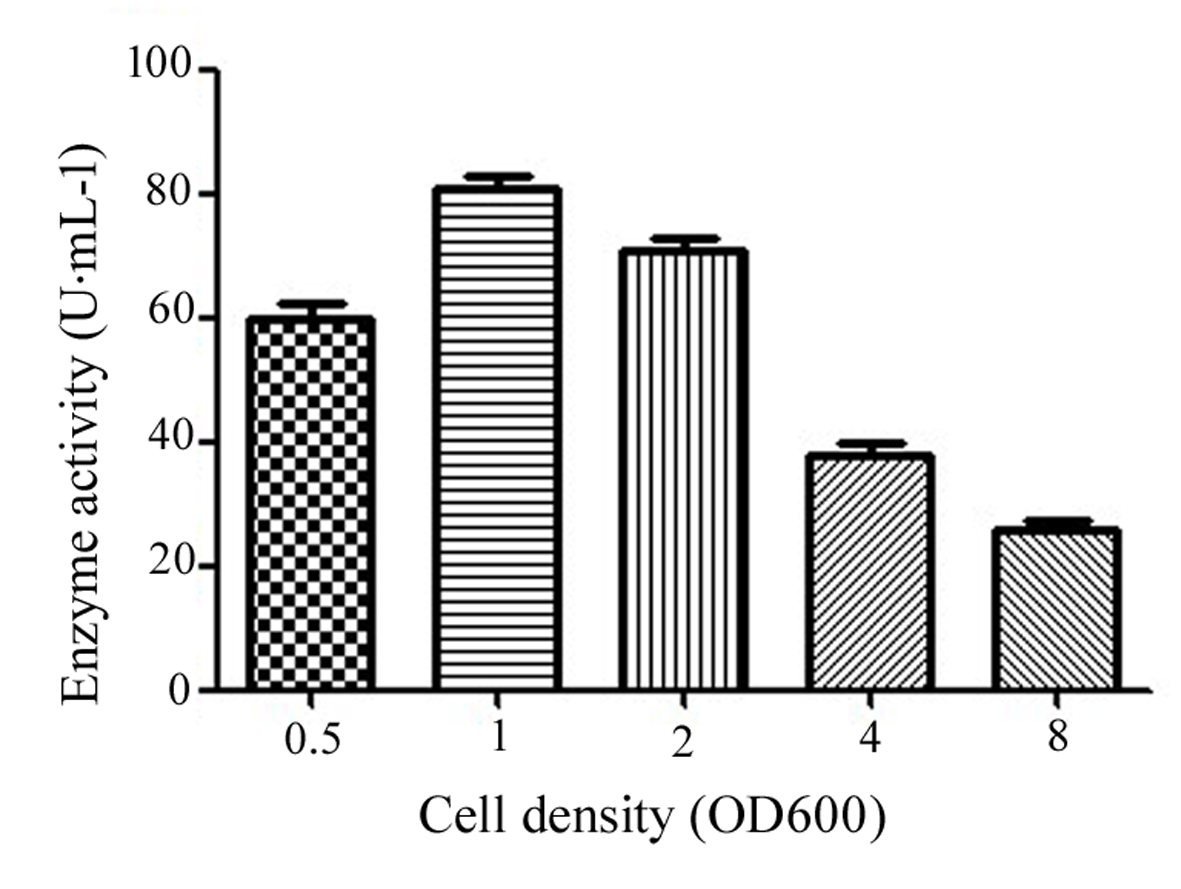
**

Figure S1. Effects of different cell density OD600 in the induction cultivation phase on the extracellular activity in the shake flasks with TB medium at 0.5,1, 2, 4, and 8.


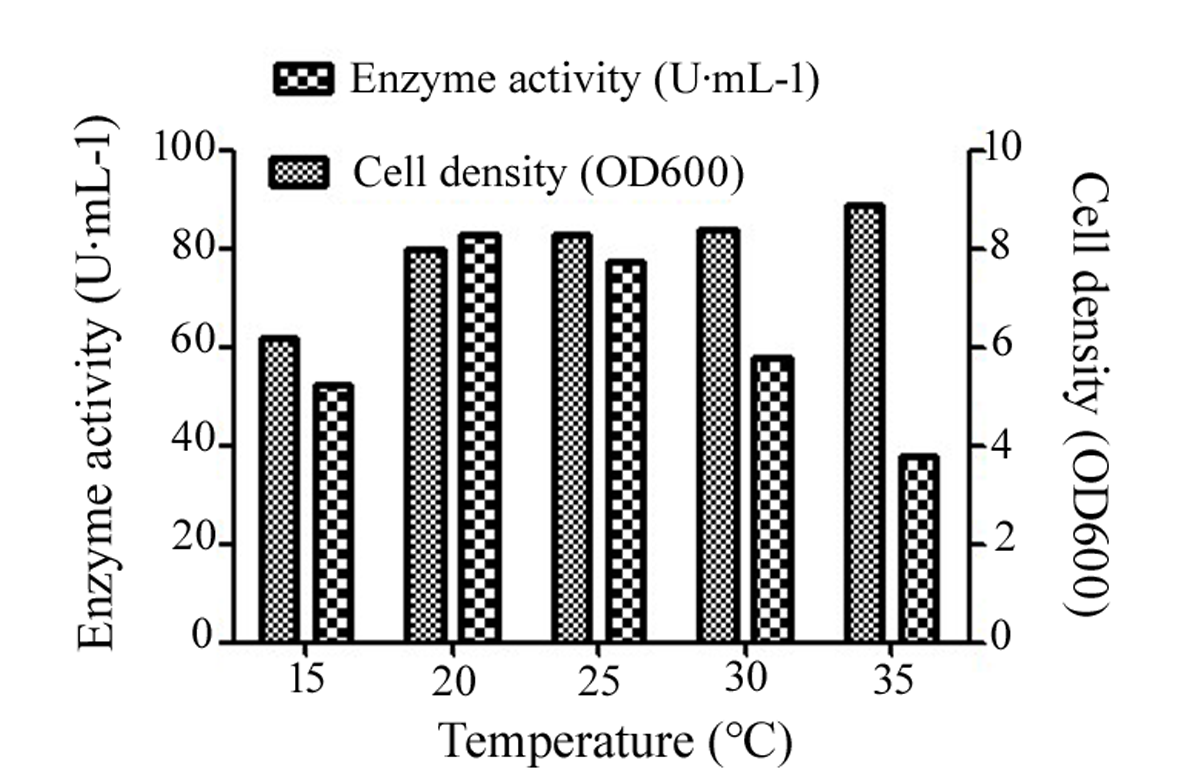


Figure S2. Effects of temperature on the extracellular activity and cell growth *Escherichia coli* strain which was grown in the shake flasks with TB medium at 15,20, 25, 30, and 35 ℃.


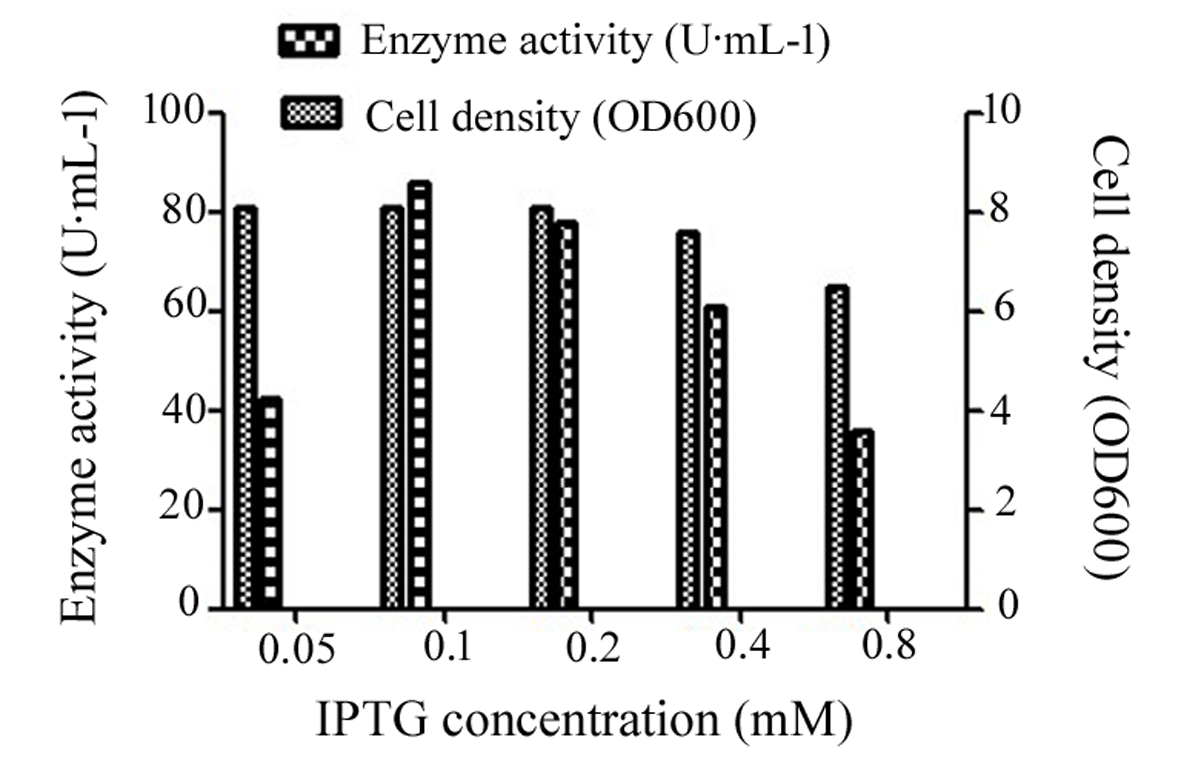


Figure S3. Effects of different concentrations of IPTG on the extracellular activity and cell growth in *Escherichia coli* strain which was grown in the shake flasks with TB medium at 0.05, 0.1, 0.2, 0.4, and 0.8mM.
